# TRANSFORMING BRAIN HEALTH IN RURAL COMMUNITIES IN WASHINGTON STATE

**DOI:** 10.1093/geroni/igae098.1225

**Published:** 2024-12-31

**Authors:** Meghan Fadel, Marci Getz, Angela Ward

**Affiliations:** Alzheimer’s Association, Chicago, Illinois, United States; Washington State Department of Health, Olympia, Washington, United States; Northeast Tri County Health District, Colville, Washington, United States

## Abstract

Rural communities experience unique strengths and barriers as they work to address dementia, making strategic action on brain health and caregiving particularly important. Since 2005, the Healthy Brain Initiative (HBI) has been working to support public health strategies that promote brain health, address dementia, and support individuals living with dementia and their caregivers in all communities. As a part of the HBI effort, the Road Map Series has served as a basis for action in rural communities by guiding local, state, and tribal public health leaders on strategies as they prioritize actions to address brain health and caregiving. The HBI also provides resources to aid in evaluation and implementation of public health approaches. One such effort is the annual Road Map Strategists Program, which helps build the capacity of local health departments and tribal organizations to address brain health. Examples of other tools include an implementation guide, a guide for health improvement planning, and an evaluation toolkit. Washington State has taken great strides in addressing brain health. Efforts include local health department participation in the HBI Road Map Strategists Program, federally funded action through a Building Our Largest Infrastructure Dementia (BOLD) Infrastructure for Alzheimer’s grant, and several initiatives led by the Washington State Dementia Action Collaborative. Lessons learned from implementing the HBI Road Map and prioritizing strategic action in rural communities and tribal nations offer valuable models for expanding work in Washington and beyond.

